# Supplementary material for: The Effects of Storage Temperature, Light Illumination, and Low-Temperature Plasma on Fruit Rot and Change in Quality of Postharvest Gannan Navel Oranges
Source: Foods. 2022 Nov 18;11(22):3707. doi: 10.3390/foods11223707 (PMC9689076; doi:10.3390/foods11223707)
Supplement: Supplementary file 1 [file foods-11-03707-s001.zip › foods-1978821-supplementary.pdf]

Supplementary Files

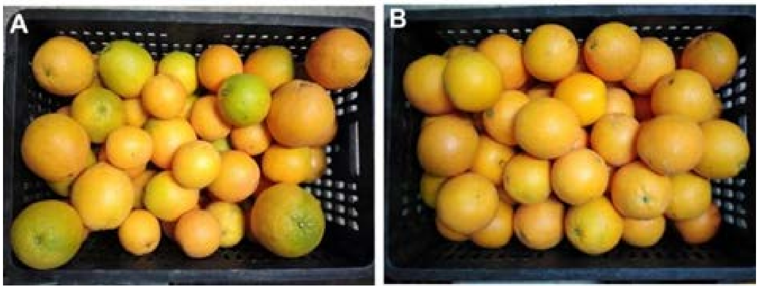

Figure S1. Fruit selection for storage experiment. **A**, fruit that were not satisfied the experimental requirements in color and size. **B**, fruit with similar color and size and no surface damage were then selected for the storage experiments.

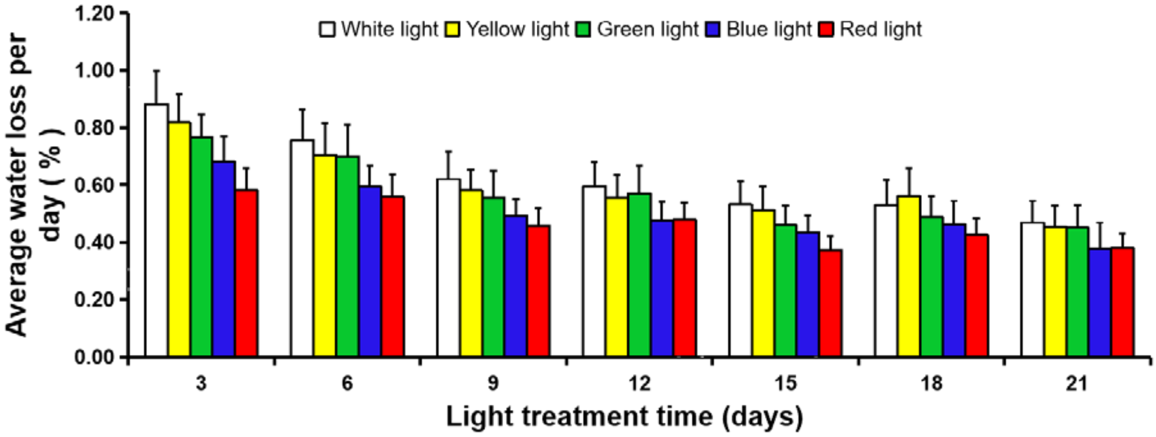

Figure S2. The average water loss (%) of single fruit under five LED light treatment at different storage time. The error bars represent the mean ± SD (n = 10).

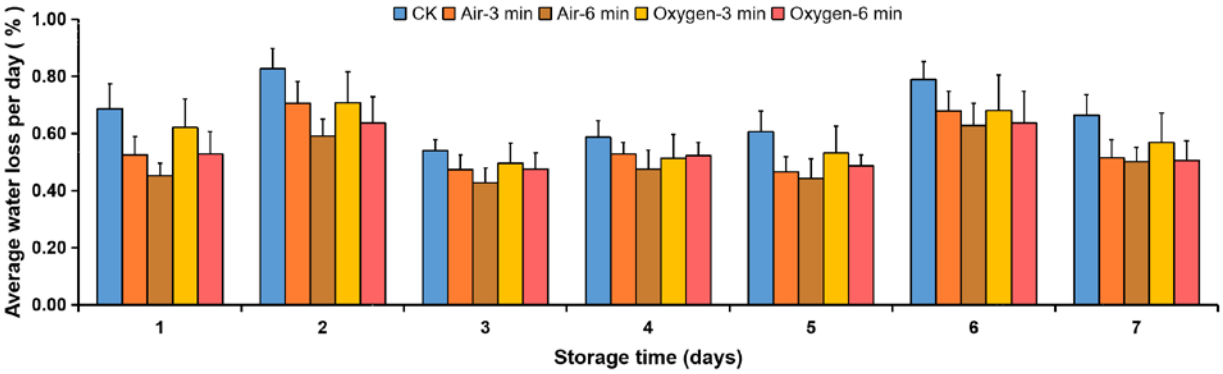

Figure S3. The average water loss (%) of single fruit of navel oranges treated by low-temperature plasma produced by air and oxygen ionization. The error bars represent the mean ± SD (n = 10).

Table S1. 1. The average water loss (%) of single fruit during storage at 22 °C.

Number	1-6 d	7-12 d	13-18 d	19-24 d	25-30 d
No.1	0.457	0.427	0.319	0.316	0.323
No.2	0.490	0.387	0.323	0.323	0.338
No.3	0.386	0.356	0.302	0.271	0.294
No.4	0.407	0.383	0.309	0.281	0.312
No.5	0.479	0.378	0.306	0.278	0.308
No.6	0.440	0.396	0.349	0.321	0.340
No.7	0.406	0.351	0.265	0.271	0.276
No.8	0.488	0.444	0.325	0.333	0.382
No.9	0.493	0.422	0.340	0.341	0.357
No.10	0.473	0.424	0.323	0.321	0.431
No.11	0.473	0.356	0.279	0.265	0.306
No.12	0.426	0.480	0.366	0.334	0.361
No.13	0.495	0.439	0.360	0.284	0.329
No.14	0.432	0.407	0.310	0.314	0.326
No.15	0.400	0.403	0.290	0.256	0.286
No.16	0.402	0.368	0.299	0.282	0.309
No.17	0.532	0.464	0.307	0.340	0.323
No.18	0.346	0.316	0.238	0.223	0.266
No.19	0.391	0.358	0.277	0.251	0.270
No.20	0.450	0.387	0.346	0.276	0.339
Mean	0.443	0.397	0.312	0.294	0.324
SD	0.047	0.042	0.032	0.034	0.040

SD: standard deviation

Table S1. 2 The average water loss (%) of single fruit during storage at 15 °C.

Number	1-6 d	7-12 d	13-18 d	19-24 d	25-30 d
No.1	0.332	0.240	0.226	0.192	0.175
No.2	0.375	0.288	0.221	0.184	0.142
No.3	0.332	0.264	0.228	0.206	0.187
No.4	0.332	0.277	0.270	0.234	0.198
No.5	0.371	0.274	0.240	0.201	0.154
No.6	0.384	0.264	0.239	0.199	0.178
No.7	0.393	0.321	0.282	0.209	0.187
No.8	0.345	0.253	0.235	0.201	0.167
No.9	0.356	0.322	0.241	0.202	0.138
No.10	0.399	0.339	0.271	0.283	0.242
No.11	0.330	0.289	0.245	0.205	0.162
No.12	0.309	0.277	0.213	0.210	0.168
No.13	0.418	0.276	0.257	0.241	0.185
No.14	0.310	0.260	0.230	0.189	0.143
No.15	0.343	0.245	0.245	0.199	0.148
No.16	0.412	0.330	0.291	0.237	0.199
No.17	0.330	0.214	0.211	0.211	0.188
No.18	0.311	0.258	0.236	0.196	0.134
No.19	0.354	0.291	0.233	0.229	0.191
No.20	0.315	0.217	0.216	0.157	0.140
Mean	0.353	0.275	0.242	0.209	0.171
SD	0.035	0.034	0.022	0.026	0.027

SD: standard deviation.

Table S1. 3 The average water loss (%) of single fruit during storage at 5 °C.

Number	1-6 d	7-12 d	13-18 d	19-24 d	25-30 d
No.1	0.213	0.205	0.171	0.160	0.156
No.2	0.267	0.261	0.224	0.220	0.232
No.3	0.257	0.262	0.226	0.202	0.223
No.4	0.171	0.176	0.151	0.149	0.148
No.5	0.211	0.220	0.194	0.169	0.178
No.6	0.196	0.214	0.173	0.180	0.193
No.7	0.189	0.203	0.168	0.156	0.164
No.8	0.193	0.198	0.163	0.165	0.159
No.9	0.151	0.160	0.142	0.132	0.138
No.10	0.173	0.178	0.146	0.136	0.146
No.11	0.181	0.181	0.179	0.173	0.173
No.12	0.164	0.161	0.149	0.132	0.149
No.13	0.213	0.210	0.184	0.166	0.168
No.14	0.256	0.265	0.238	0.229	0.230
No.15	0.242	0.246	0.214	0.205	0.209
No.16	0.191	0.201	0.185	0.177	0.180
No.17	0.210	0.207	0.188	0.172	0.184
No.18	0.206	0.205	0.183	0.183	0.176
No.19	0.174	0.170	0.162	0.147	0.158
No.20	0.162	0.181	0.163	0.152	0.162
Mean	0.201	0.205	0.180	0.170	0.176
SD	0.033	0.032	0.028	0.027	0.028

SD: standard deviation.

Table S2. 1 The average water loss (%) of single fruit under white LED light illumination at different storage time.

Number	3 d	6 d	9 d	12 d	15 d	18 d	21d
No.1	0.78	0.63	0.52	0.50	0.47	0.42	0.39
No.2	0.84	0.66	0.52	0.49	0.42	0.44	0.37
No.3	1.16	1.00	0.85	0.78	0.69	0.72	0.60
No.4	0.86	0.74	0.63	0.56	0.51	0.53	0.45
No.5	0.86	0.74	0.61	0.61	0.51	0.52	0.45
No.6	0.80	0.70	0.60	0.55	0.49	0.48	0.40
No.7	0.79	0.72	0.56	0.57	0.49	0.47	0.42
No.8	0.86	0.74	0.60	0.63	0.57	0.57	0.55
No.9	0.86	0.75	0.59	0.59	0.53	0.56	0.51
No.10	0.99	0.87	0.71	0.67	0.63	0.60	0.53
Mean	0.88	0.76	0.62	0.59	0.53	0.53	0.47
SD	0.11	0.11	0.10	0.08	0.08	0.09	0.08

SD: standard deviation.

Table S2. 2 The average water loss (%) of single fruit under yellow LED light illumination at different storage time.

Number	3 d	6 d	9 d	12 d	15 d	18 d	21d
No.1	1.03	0.93	0.75	0.74	0.66	0.74	0.56
No.2	0.79	0.65	0.54	0.55	0.46	0.49	0.40
No.3	0.83	0.72	0.59	0.57	0.52	0.53	0.43
No.4	0.76	0.60	0.51	0.49	0.42	0.50	0.39
No.5	0.80	0.67	0.57	0.54	0.49	0.50	0.41
No.6	0.79	0.70	0.55	0.51	0.53	0.51	0.39
No.7	0.66	0.55	0.51	0.47	0.40	0.44	0.41
No.8	0.76	0.67	0.56	0.50	0.48	0.57	0.44
No.9	0.86	0.74	0.60	0.55	0.57	0.63	0.52
No.10	0.89	0.84	0.63	0.64	0.59	0.69	0.59
Mean	0.82	0.71	0.58	0.56	0.51	0.56	0.45
SD	0.10	0.11	0.07	0.08	0.08	0.10	0.07

SD: standard deviation.

Table S2. 3 The average water loss (%) of single fruit under green LED light illumination at different storage time.

Number	3 d	6 d	9 d	12 d	15 d	18 d	21d
No.1	0.79	0.68	0.54	0.55	0.46	0.47	0.44
No.2	0.64	0.58	0.46	0.44	0.34	0.36	0.31
No.3	0.72	0.66	0.52	0.53	0.43	0.44	0.40
No.4	0.76	0.66	0.53	0.54	0.44	0.46	0.39
No.5	0.92	0.94	0.79	0.78	0.60	0.64	0.61
No.6	0.76	0.63	0.50	0.54	0.43	0.51	0.49
No.7	0.81	0.77	0.60	0.65	0.50	0.52	0.50
No.8	0.83	0.81	0.62	0.64	0.49	0.51	0.46
No.9	0.69	0.61	0.49	0.48	0.42	0.46	0.41
No.10	0.75	0.66	0.53	0.56	0.48	0.52	0.48
Mean	0.77	0.70	0.56	0.57	0.46	0.49	0.45
SD	0.08	0.11	0.09	0.10	0.07	0.07	0.08

SD: standard deviation.

Table S2. 4 The average water loss (%) of single fruit under blue LED light illumination at different storage time.

Number	3 d	6 d	9 d	12 d	15 d	18 d	21d
No.1	0.79	0.62	0.56	0.50	0.38	0.45	0.36
No.2	0.67	0.64	0.50	0.44	0.41	0.46	0.33
No.3	0.79	0.75	0.58	0.56	0.47	0.49	0.41
No.4	0.81	0.62	0.56	0.57	0.51	0.63	0.58
No.5	0.64	0.54	0.46	0.43	0.49	0.40	0.33
No.6	0.56	0.52	0.40	0.40	0.32	0.40	0.29
No.7	0.67	0.63	0.50	0.55	0.48	0.57	0.47
No.8	0.58	0.50	0.43	0.41	0.37	0.38	0.31
No.9	0.67	0.55	0.47	0.48	0.48	0.43	0.38
No.10	0.64	0.55	0.47	0.43	0.41	0.41	0.31
Mean	0.68	0.59	0.49	0.48	0.43	0.46	0.38
SD	0.09	0.07	0.06	0.07	0.06	0.08	0.09

SD: standard deviation.

Table S2. 5 The average water loss (%) of single fruit under red LED light illumination at different storage time.

Number	3 d	6 d	9 d	12 d	15 d	18 d	21d
No.1	0.75	0.73	0.59	0.60	0.48	0.53	0.45
No.2	0.56	0.54	0.44	0.49	0.40	0.46	0.44
No.3	0.58	0.54	0.43	0.47	0.35	0.41	0.39
No.4	0.57	0.55	0.42	0.44	0.33	0.37	0.34
No.5	0.59	0.55	0.45	0.51	0.40	0.47	0.43
No.6	0.50	0.50	0.42	0.45	0.35	0.39	0.33
No.7	0.60	0.56	0.47	0.47	0.37	0.43	0.38
No.8	0.67	0.66	0.53	0.54	0.39	0.48	0.40
No.9	0.51	0.49	0.40	0.43	0.33	0.37	0.32
No.10	0.51	0.47	0.41	0.40	0.31	0.35	0.31
Mean	0.58	0.56	0.46	0.48	0.37	0.43	0.38
SD	0.08	0.08	0.06	0.06	0.05	0.06	0.05

SD: standard deviation.

Table S3. 1 The average water loss (%) of single fruit of navel oranges treated by low-temperature plasma produced by oxygen ionization 3 min.

Number	1 d	2 d	3 d	4 d	5 d	6 d	7 d
No.1	0.59	0.58	0.46	0.46	0.50	0.76	0.54
No.2	0.73	0.76	0.54	0.64	0.67	0.85	0.65
No.3	0.52	0.70	0.43	0.42	0.53	0.54	0.56
No.4	0.65	0.81	0.55	0.49	0.50	0.71	0.69
No.5	0.57	0.55	0.47	0.58	0.48	0.58	0.61
No.6	0.69	0.82	0.51	0.55	0.59	0.69	0.48
No.7	0.47	0.59	0.38	0.40	0.35	0.57	0.38
No.8	0.58	0.75	0.50	0.53	0.60	0.57	0.54
No.9	0.59	0.67	0.48	0.44	0.45	0.64	0.51
No.10	0.81	0.85	0.64	0.61	0.64	0.89	0.72
Mean	0.62	0.71	0.50	0.51	0.53	0.68	0.57
SD	0.10	0.11	0.07	0.08	0.09	0.12	0.10

SD: standard deviation.

Table S3. 2 The average water loss (%) of single fruit of navel oranges treated by low-temperature plasma produced by oxygen ionization 6 min.

Number	1 d	2 d	3 d	4 d	5 d	6 d	7 d
No.1	0.42	0.57	0.41	0.44	0.43	0.51	0.44
No.2	0.57	0.63	0.48	0.58	0.53	0.72	0.59
No.3	0.51	0.70	0.51	0.51	0.52	0.71	0.46
No.4	0.51	0.60	0.43	0.52	0.44	0.70	0.60
No.5	0.58	0.73	0.43	0.56	0.44	0.65	0.43
No.6	0.48	0.64	0.49	0.56	0.52	0.67	0.44
No.7	0.58	0.50	0.48	0.52	0.48	0.66	0.49
No.8	0.68	0.81	0.61	0.57	0.51	0.79	0.61
No.9	0.48	0.62	0.43	0.47	0.47	0.44	0.49
No.10	0.45	0.55	0.47	0.50	0.50	0.53	0.51
Mean	0.53	0.64	0.47	0.52	0.49	0.64	0.51
SD	0.08	0.09	0.06	0.05	0.04	0.11	0.07

SD: standard deviation.

Table S3. 3 The average water loss (%) of single fruit of navel oranges treated by low-temperature plasma produced by air ionization 3 min.

Number	1 d	2 d	3 d	4 d	5 d	6 d	7 d
No.1	0.53	0.74	0.53	0.56	0.42	0.71	0.51
No.2	0.43	0.71	0.40	0.51	0.56	0.75	0.52
No.3	0.48	0.74	0.49	0.57	0.45	0.70	0.52
No.4	0.46	0.69	0.44	0.55	0.45	0.65	0.62
No.5	0.54	0.76	0.46	0.47	0.42	0.66	0.41
No.6	0.54	0.61	0.51	0.54	0.52	0.59	0.50
No.7	0.55	0.65	0.51	0.55	0.52	0.67	0.56
No.8	0.53	0.65	0.41	0.46	0.48	0.63	0.54
No.9	0.67	0.87	0.55	0.57	0.42	0.81	0.55
No.10	0.51	0.62	0.43	0.51	0.42	0.60	0.42
Mean	0.52	0.70	0.47	0.53	0.47	0.68	0.51
SD	0.07	0.08	0.05	0.04	0.05	0.07	0.06

SD: standard deviation.

Table S3. 4 The average water loss (%) of single fruit of navel oranges treated by low-temperature plasma produced by air ionization 6 min.

Number	1 d	2 d	3 d	4 d	5 d	6 d	7 d
No.1	0.41	0.57	0.47	0.41	0.40	0.69	0.52
No.2	0.43	0.62	0.44	0.50	0.46	0.55	0.48
No.3	0.50	0.64	0.37	0.48	0.30	0.56	0.49
No.4	0.38	0.63	0.38	0.41	0.42	0.58	0.54
No.5	0.47	0.58	0.41	0.55	0.51	0.70	0.53
No.6	0.48	0.57	0.52	0.52	0.54	0.73	0.51
No.7	0.42	0.50	0.43	0.37	0.41	0.48	0.40
No.8	0.48	0.62	0.37	0.46	0.48	0.65	0.49
No.9	0.52	0.69	0.48	0.59	0.50	0.65	0.59
No.10	0.42	0.50	0.39	0.45	0.39	0.67	0.45
Mean	0.45	0.59	0.43	0.47	0.44	0.63	0.50
SD	0.04	0.06	0.05	0.07	0.07	0.08	0.05

SD: standard deviation.
